# Canadian Arctic sea ice reconstructed from bromine in the Greenland NEEM ice core

**DOI:** 10.1038/srep33925

**Published:** 2016-09-21

**Authors:** Andrea Spolaor, Paul Vallelonga, Clara Turetta, Niccolò Maffezzoli, Giulio Cozzi, Jacopo Gabrieli, Carlo Barbante, Kumiko Goto-Azuma, Alfonso Saiz-Lopez, Carlos A. Cuevas, Dorthe Dahl-Jensen

**Affiliations:** 1 Ca’Foscari University of Venice, Department of Environmental Science, Informatics and Statistics, Via Torino 155, 30172 Mestre, Venice, Italy; 2 Institute for the Dynamics of Environmental Processes, IDPA-CNR, Via Torino 155, 30172 Mestre, Venice, Italy; 3 Centre for Ice and Climate, Niels Bohr Institute, University of Copenhagen, Juliane Maries Vej 30, Copenhagen Ø 2100 Denmark; 4 National Institute of Polar Research, 10-3 Midori-cho, Tachikawa Tokyo, 190-8518, Japan; 5 Department of Atmospheric Chemistry and Climate, Institute of Physical Chemistry Rocasolano, CSIC, Serrano 119, 28006 Madrid, Spain

## Abstract

Reconstructing the past variability of Arctic sea ice provides an essential context for recent multi-year sea ice decline, although few quantitative reconstructions cover the Holocene period prior to the earliest historical records 1,200 years ago. Photochemical recycling of bromine is observed over first-year, or seasonal, sea ice in so-called “bromine explosions” and we employ a 1-D chemistry transport model to quantify processes of bromine enrichment over first-year sea ice and depositional transport over multi-year sea ice and land ice. We report bromine enrichment in the Northwest Greenland Eemian NEEM ice core since the end of the Eemian interglacial 120,000 years ago, finding the maximum extension of first-year sea ice occurred approximately 9,000 years ago during the Holocene climate optimum, when Greenland temperatures were 2 to 3 °C above present values. First-year sea ice extent was lowest during the glacial stadials suggesting complete coverage of the Arctic Ocean by multi-year sea ice. These findings demonstrate a clear relationship between temperature and first-year sea ice extent in the Arctic and suggest multi-year sea ice will continue to decline as polar amplification drives Arctic temperatures beyond the 2 °C global average warming target of the recent COP21 Paris climate agreement.

A seasonal cycle in polar atmospheric bromine was first discovered in the 1980s by Sturges and Barrie^1^ with higher bromine concentrations observed during Arctic spring. Subsequent studies^2^,^3^ have established the photochemical nature of bromine atmospheric chemistry and its reaction with tropospheric ozone^4^,^5^ to form bromine oxide (BrO)^5^ (see Supplementary Information for a detailed chemical description). Satellite sensors associate enhanced BrO concentrations with first-year sea ice (FYSI) rather than multi-year sea ice (MYSI)^6^,^7^. Enhanced bromine photochemistry at the FYSI surface^8^ has been related to the presence of highly saline conditions such as those found in brine channels^3^, frost flowers^9^ and blowing snow^10^, which are not generally associated with MYSI, as most of the brine is drained away during the summer melt^11^,^12^. As heterogeneous and photochemical recycling of bromine (bromine explosion) is associated with marginal sea ice regions, bromine deposited on land ice is enriched relative to sea salt compositions at coastal locations^13^ and progressively depleted as airmasses travel further inland^14^.

An understanding of polar tropospheric halogen chemistry has developed as ground- and ice-core based measurements have established clearer links between FYSI extent and halogen variability recorded in polar ice cores. Interactions between halogens and FYSI are monitored by satellite^6^ and ground-based^7^ sensors, while interactions with snowpack are a topic of active investigation^8^,^15^,^16^. Seasonal variability in bromine enrichment has been observed in surface snow samples from Antarctica (Law Dome^17^) and the Arctic (Northwest Greenland^17^, Svalbard^18^, Severnaya Zemlya^19^). Such enrichment beyond seawater compositions consistently occurs in spring/summer and is consistent with photochemical recycling processes linked to FYSI. A fifty-year record of bromine enrichment and iodine from Severnaya Zemlya is well correlated with observations of Laptev sea FYSI extent^19^. Bromine enrichment variations observed in the Antarctic Talos Dome ice core agree well with reconstructions of sea ice duration based on fossil diatom assemblages from a marine sediment core over the last two glacial/interglacial cycles^14^. This study presents the first record of bromine in the Arctic covering the last glacial cycle.

Due to the poor geographical and temporal resolution of sea ice proxies, few quantitative reconstructions of Holocene Arctic total sea ice extent are available. The few reconstructions available are based on dinoflagellate cyst assemblages^20^, specific highly branched isoprenoid monoenes (IP_25_) derived from sea ice diatoms^21^, melt layers in ice cores^22^ as well as driftwood remains on raised beaches^23^. Reconstructions of Arctic MYSI describe a broad pattern of minimal MYSI extent during warmer climate phases such as the early Holocene (8–11.7 ky before the year 2000 hereafter referred to as ‘b2k’) and Bølling-Allerød (12.9–14.7 ky b2k)^23^, contrasting with greater MYSI extent during cooler climate phases such as the late Holocene (0–3 ky b2k) and extensive MYSI extent during the last glacial maximum (14.7–23 ky b2k)^24^. MYSI variability broadly follows northern latitude summer insolation, with a minimum of Arctic MYSI cover in the Canadian Arctic during the early Holocene (8–11.7 ky ago)^13^. Frequent, high-amplitude fluctuations in MYSI extent are apparent since 5 ky b2k and particularly over the last 1,450 years, for which well-resolved multi-proxy reconstructions of MYSI extent with good geographical coverage are available^25^. Rapid fluctuations in iceberg export through the Fram Strait during the Younger Dryas (11.7–12.9 ky BP) and Bølling-Allerød have been reconstructed^26^,^27^ from ocean sediment cores recovered from sites featuring high sedimentation rates.

The drivers and feedbacks between polar climate and total sea ice remain a topic of active research. The ice- albedo feedback mechanism has been studied in detail^28^ and is particularly relevant to decreasing MYSI in the last two decades as well as during abrupt climate changes such as the onset of Greenland interstadial events. Mechanisms controlling Arctic MYSI variability during the glacial are a topic of speculation, with a recent study proposing the formation of warm subsurface Atlantic water as a precursor and driver of rapid MYSI collapse in the Nordic seas^29^. Possible influences of North Atlantic deepwater formation as well as Atlantic meridional overturning circulation have been also been considered^30^. Ice core-based reconstructions of precipitation moisture sources, dust transport and sea salt proxies^31^ indicate large-scale atmospheric and oceanic circulation changes occurring through the Bølling-Allerød and Younger Dryas glacial termination sequence and point to a key role for changes in North Atlantic Ocean surface conditions^32^.

Here we present bromine and sodium concentrations in the NEEM ice core to investigate Canadian Arctic sea ice variability since the last interglacial period. We define the Canadian Arctic as the area from which aerosols are entrained and transported to the NEEM site, comprising the Canadian archipelago, Baffin Bay and Hudson Bay regions (see Supplementary Information). Bromine is proposed as a FYSI proxy in ice cores^14^,^18^,^19^, supported by halogen chemistry transport modelling which demonstrates the effectiveness of bromine recycling over FYSI and subsequent bromine depletion with transport. Back trajectory modelling indicates the majority of airmasses originate from the west and arrive at NEEM after entraining air primarily from the Baffin Bay, Hudson Bay and Canadian Arctic regions. The study of bromine in polar ice cores offers a new technique to produce quantitative reconstructions of FYSI and MYSI of unprecedented temporal resolution.

## Results

### NEEM ice core

Bromine and sodium concentrations have been determined in the North Greenland Eemian NEEM ice core^33^ (North Greenland Eemian Ice Drilling Project, 77°45′ N, 51°06′ W) located in northwest Greenland at an altitude of 2,484 m a.s.l. The core offers a detailed archive of the Holocene and last glacial period as well as allowing the first complete record of Greenland climate during the Eemian interglacial. Snow deposited at NEEM originates primarily from the south-west with long-range aerosol transport associated with emission sources in northern North America and Asia^34^,^35^ (see Supplementary Information). Marine aerosols in northwest Greenland originate from the Canadian Arctic and Baffin Bay sectors^36^ and as such are considered the dominant source locations of Br deposited at the NEEM site. Samples were collected at irregular depth intervals although each sample integrates a 1.10 m depth range. Due to the thinning of annual layers with depth, each sample integrates 4 to 20 years in the Holocene and 60 to 120 years in the glacial period (see Supplementary material). Sampling was concentrated in the depth range of Greenland stadials/interstadials (GSs/GIs) 8 to 13^37^. Samples covering the period 3 to 9 ky b2k were unavailable for analysis due to the poor quality of the core in the “brittle ice” section where trapped gas bubbles are transformed into clathrates.

### Bromine concentrations

**Figure 1:**
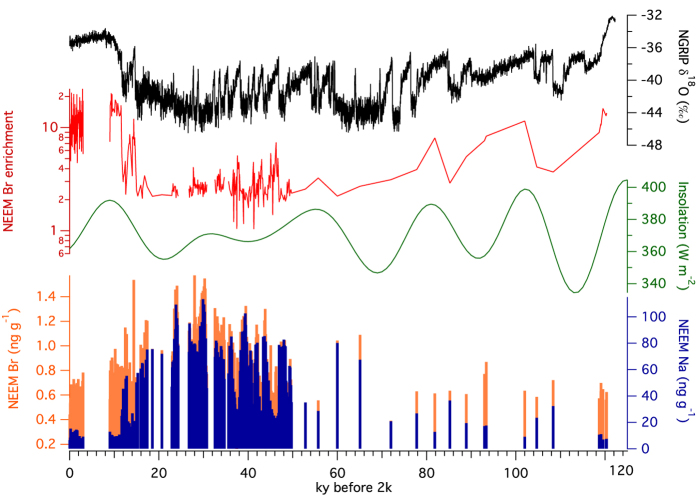
Bromine and sodium in the NEEM ice core over the past glacial cycle. Bromine and sodium concentrations are decoupled from interglacial to glacial climates. The enrichment of bromine relative to sodium (Br enrichment) more closely follows the NGRIP δ^18^O temperature proxy rather than 65°N summer solstice insolation^45^. Minimum Br_enr_ values are observed during the coldest phases of the glacial, while the maximum is observed during the Holocene climatic optimum.

Bromine concentrations in the NEEM ice core record vary through the Holocene as well as during the rapid interstadial warming events (Fig. 1). The lowest bromine concentrations are observed during the late Holocene (0.47 ± 0.15 ppb (1σ) 0–3 ky b2k) with higher concentrations during the early Holocene (0.80 ± 0.08 ppb (1σ) 9–11.5 ky b2k) and the highest observed concentrations during the late glacial stadials (eg. 1.45 ± 0.07 ppb (1σ) 29.6–30.4 ky b2k). Sodium shows similar variability although it is notable that sodium concentrations are proportionally lower during the Holocene, leading to greater variability in the bromine enrichment and enhancing the distinction between sea-salt bromine and photochemically enriched bromine. In order to quantify the influence of bromine enrichment resulting from sea ice-related bromine explosion events, we calculate Br_enr_ as Br/(Na*0.006) using the mass ratios of Br and Na and the average sea water Br/Na mass ratio (0.006^38^). Although bromine concentrations are negatively correlated with δ^18^O temperature proxy (R^2^ = 0.50), Br_enr_ is positively correlated with δ^18^O (R^2^ = 0.54) supporting the connection between warm climate conditions, greater presence of FYSI and enhanced bromine photochemistry.

### Chemistry transport model

**Figure 2:**
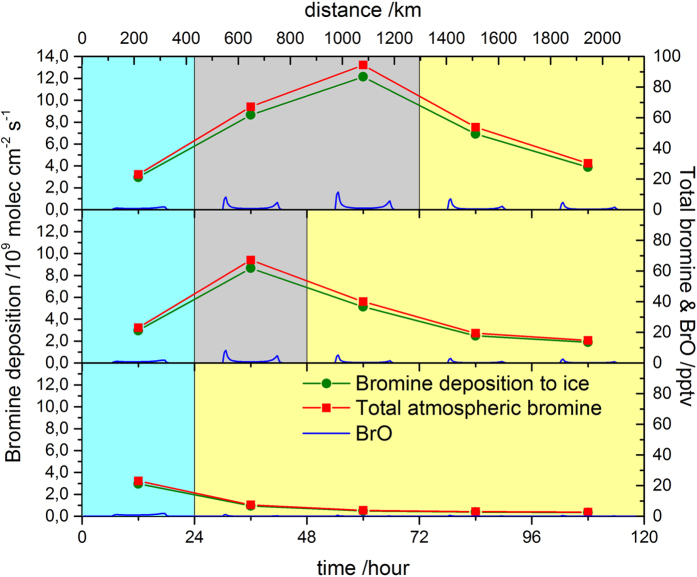
Simulated diurnal averages of total atmospheric bromine and deposited bromine, along with gas phase BrO mixing ratios. Three simulations were run to illustrate the sensitivity of bromine deposition at NEEM to distant “bromine explosion” events. In each case, the air mass initially spends 24 hours over open ocean (blue). (**a**) Upper panel shows 48 hours over FYSI (grey) with active bromine recycling on the surface, and 48 hours over MYSI/Greenland (yellow). (**b**) Middle panel shows 24 hours over FYSI (grey) with active bromine recycling on the surface, and 72 hours over MYSI/Greenland (yellow). (**c**) Lower panel shows the simulated air mass without encountering FYSI.

To demonstrate the quantitative link between FYSI extent and bromine enrichment in the NEEM ice core, we have applied a 1-dimensional setup of a state-of-the-art chemistry transport model featuring interactive halogen photochemistry. The model is spun up with a constant atmospheric BrO concentration over open ocean and then transports the airmass over two types of surface: FYSI over which bromine is photochemically recycled; and a stable surface over which bromine is not recycled. This stable non-reactive surface is theorised to comprise a combination of MYSI and/or Greenland ice sheet surface (land ice). Three scenarios have been investigated, in which the airmass experiences bromine recycling for 48 hours, 24 hours, or not at all (Fig. 2). This final scenario corresponds to an absence of FYSI in the Arctic basin and instead complete MYSI cover (see Supplementary Information). The model demonstrates that bromine is enriched in the air mass for at least 72 hours after recycling over FYSI. Assuming a moderate velocity of 5 m s^−1^, a bromine-enriched air mass would travel more than 1,000 km in 72 hours, easily encompassing the distance from the Canadian Arctic to the NEEM site. In the absence of recycling, atmospheric bromine concentrations quickly decrease to levels an order of magnitude lower than those found in the FYSI scenarios. On the basis of the model results, we proceed with the interpretation that bromine enhancement at the NEEM site is quantitatively linked to the presence of FYSI and that bromine concentrations found at NEEM are reasonably independent of transport variability. This interpretation assumes explicitly that no bromine recycling occurs over MYSI or land ice, and that bromine enrichment may be employed as a quantitative indicator of bromine recycling over FYSI.

## Discussion

**Figure 3:**
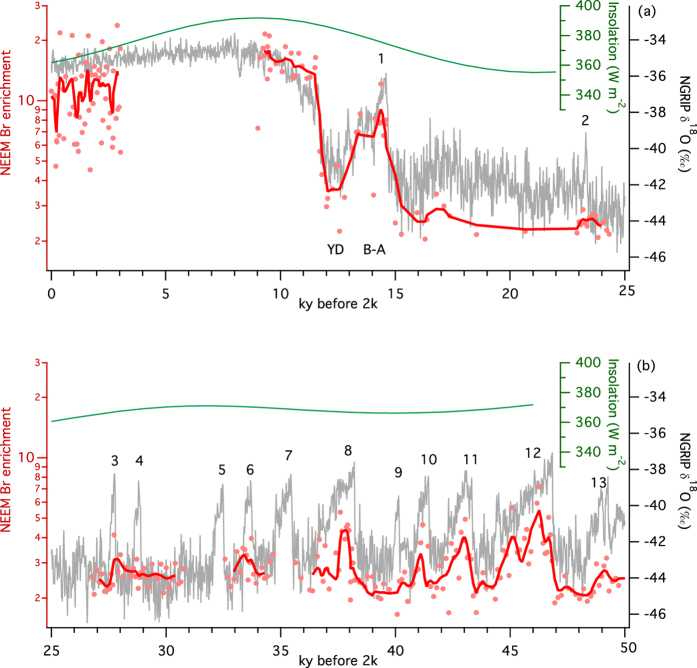
Variability in bromine enrichment occurs on stadial-interstadial scales. Bromine enrichment follows the NGRIP δ^18^O temperature paleoproxy profile during abrupt climate changes in the glacial. The greatest Br_enr_ values observed in the Holocene (panel a) occur during the Holocene Climate Optimum and not in the most recent samples. In the glacial (panel b), consistently lower Br_enr_ values are observed, corresponding to a greater extent of multi-year sea ice in the Canadian Arctic. Bromine enrichment does not closely follow 65°N summer solstice insolation^45^. Note the scales are identical in both panels.

Bromine enrichment in the NEEM ice core is greatest during the early Holocene, indicating a substantial increase of FYSI and corresponding decrease of MYSI in the Arctic immediately after the glacial termination (Fig. 3a). Ice-core based temperature reconstructions indicate Greenland was warmer than present by 2 to 3 °C during the Holocene Climatic Optimum, broadly assigned to the period 6–9 ky ago, when summer insolation at high northern latitudes was at a maximum^39^. Evidence from marine sediment biomarkers, ice core melt and mammalian remains demonstrate warmer Arctic conditions and a minimum of MYSI in the Canadian Arctic at that time^13^,^27^. Early Holocene Bromine enrichments (Br_enr_ 15.6 ± 3.2 (1σ) 9.0–11.5 ky b2k), associated with the prevalence of FYSI, support existing evidence of a rapid loss of MYSI at the end of the glacial and a relatively ice-free Arctic Ocean. In comparison, bromine enrichment is lower during the last 3 ky (Br_enr_ 10.6 ± 4.9 (1σ) 0.03–3.03 ky b2k) and displays greater variability, suggesting greater extension of MYSI in the Canadian Arctic during the late Holocene. Expansion of MYSI in recent millennia is supported by reconstructions of Arctic sea ice determined from Arctic Ocean marine sediments^40^ and driftwood deposition on paleo-beach ridges^23^.

Over the last glacial period, bromine enrichment shows variability on the scales of Dansgaard-Oeschger (DO) events. DO events are rapid climate changes involving reorganisation of atmospheric and oceanic circulation on hemispheric and interhemispheric scales. Greenland interstadials (GI) 3 to 12 (Fig. 3b) demonstrate approximately similar magnitudes of isotopic variability, but over different durations from 0.24 ky (GI-3) to 2.58 ky (GI-12). Bromine enrichment is greatest during longer-lasting interstadials (GI-8, 10, 11, 12) and is minimal for interstadials of less than 1000 years duration (GI-3, 4, 6, 9). The combined chemistry model and NEEM data suggest the Arctic basin was completely covered by MYSI during Greenland Stadials (GSs) and during the late glacial, resulting in minimal bromine enrichment. In addition to extensive MYSI coverage of the Arctic Ocean during the glacial maximum, paleo sea level reconstructions suggest extensive land ice coverage over areas currently occupied by the Canadian Archipelago and the Beaufort, Chukchi, Barents, Kara and Laptev seas, but not over Baffin bay^41^. For the longest GIs, MYSI was partly replaced by FYSI, which allowed springtime bromine recycling and the deposition of enriched bromine at NEEM. Bromine enrichment during the Bølling-Allerød (GI-1) and Younger Dryas (GS-1) events show intermediate values between glacial and Holocene levels, with rapid changes in Br_enr_ closely following changes in δ^18^O temperature proxy. Bromine enrichment during GI-1 is similar to late Holocene values, indicating a mixture of MYSI and FYSI in the Canadian Arctic. Between 11.8 and 12.8 ky b2k, consistently low Br_enr_ values indicate a return to extensive MYSI cover in the Canadian Arctic.

**Figure 4:**
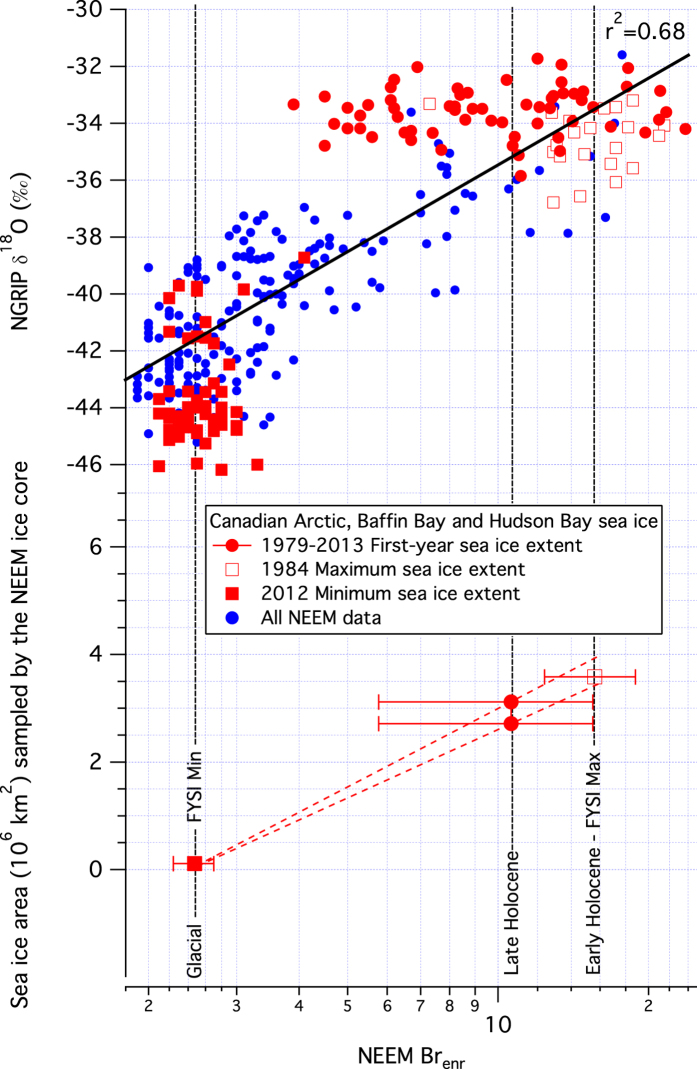
Calibration of the NEEM Br enrichment (Br_enr_) signal to current and past sea ice extent in the NEEM aerosol entrainment area (Canadian Archipelago, Hudson Bay, Baffin Bay). Three climate scenarios are evaluated, full glacial (Br_enr_ = 2.5, 20.6–30.7 ky b2k), late Holocene (Br_enr_ = 10.6, 0.03–3.03 ky b2k) and early Holocene (Br_enr_ = 15.6, 9.0–11.5 ky b2k). For each scenario, we employ the maxima and minima of sea ice extent observed between 1979 and 2013, and range of first-year sea ice variability, for calibration. Bromine enrichment is plotted on a logarithmic scale to provide the best linear fit of Arctic temperature variability represented by the NGRIP δ^18^O profile. 1σ error bars represent the variability observed for the three climate intervals evaluated.

The NEEM bromine enrichment data have been used for a quantitative evaluation of sea ice conditions in the Canadian Arctic over the Holocene. The data indicate substantial reduction in MYSI during the Holocene and particularly during the Holocene Climate Optimum from 9 to 11.5 ky ago. Under the assumptions: that the Arctic Ocean was completely covered by MYSI during the late glacial; and that the range of Canadian Arctic FYSI sea ice extent observed during the satellite era can be considered representative of late Holocene FYSI; we apply a simple linear regression to the Br_enr_ values found for glacial and late Holocene climates (Fig. 4). This simple model allows an evaluation of the FYSI extent that may have occurred during the early Holocene, when Greenland was warmer than present by 2 to 3 °C^39^. We use Br_enr_ values reported above for the late and early Holocene, and Br_enr_ = 2.5 ± 0.4 (1σ, n = 51, 20.6–30.7 ky b2k) to represent late glacial conditions (see Supplementary Information). We find that a linear regression model provides a statistically robust representation of the δ^18^O-log(Br_enr_) relationship (r^2^ = 0.68, p < 0.001) for the whole NEEM dataset presented here and therefore also use log(Br_enr_) for our linear model of FYSI response to Holocene temperature variability. The model (Fig. 4) suggests an early-Holocene FYSI extent of 3.7 ± 0.3 × 10^6^ km^2^, corresponding to a 10^6^ km^2^ increase in surface area beyond the 1979–2013 average FYSI extent of 2.7 × 10^6^ km^2^. Given that the 1979–2013 MYSI extent in the Canadian Arctic is 0.44 × 10^6^ km^2^, a FYSI increase of 10^6^ km^2^ during the early Holocene would require complete loss of MYSI in the Canadian Arctic as well as MYSI reduction in bounding sea ice zones, such as the Beaufort Sea and/or Arctic Ocean. In the context of recent Arctic MYSI decrease^42^ these data indicate that MYSI is likely to continue to decrease as Arctic conditions tend towards those last experienced during the Holocene climate optimum.

The NEEM bromine record complements marine sediment records from the Fram strait and Nordic seas, allowing the timing and geographical impacts of sea ice collapse to be evaluated with respect to Arctic warming. Bromine enrichment should be determined in ice cores from East Greenland, to provide an independent reconstruction of late-glacial MYSI activity identified in the Fram Strait^27^. Remeasurement of the NEEM glacial ice with increased chronological resolution will allow an unprecedented view of glacial climate dynamics during times of abrupt change. Bromine in ice cores overcomes the drawbacks present in existing paleo sea ice proxies, being stable for tens if not hundreds of millennia; being quantitatively linked to sea ice extent and minimally dependent on transport variability; and being capable of high temporal resolution extending back to the Eemian interglacial period.

## Methods

Samples were collected from the Continuous Flow Analysis system operated by the University of Bern at the NEEM ice core drilling site. For every 1.10 m rod of ice melted, 10 mL of meltwater was collected in an acid-cleaned polyethylene bottle and immediately frozen. The frozen samples were sent to the University of Venice for analysis. The samples were kept frozen and shielded from light until analysis.

Concentrations of Br and Na were determined by Inductively Coupled Plasma Sector Field Mass Spectrometry (ICP-SFMS; Element2, ThermoFischer, Bremen, Germany) equipped with a cyclonic Peltier-cooled spray chamber (ESI, Omaha, USA)^14^. The sample flow was maintained at 0.4 mL min^−1^. Detection limits, calculated as three times the standard deviation of the blank, were 50 pg g^−1 79^Br. Reproducibility was evaluated by repeating measurements of selected samples characterized by different concentration values (between 400 and 600 pg g^−1^ for Br). The residual standard deviation (RSD) was low for both halogens and ranged between 2–10%.

The analytical system was cleaned for 24 h prior to each analysis session, consisting of alternating 180 s washes of 5% ammonium solution (*Trace*SELECT^®^ NH_4_OH, Sigma Aldrich), then 2% HNO_3_ acid (trace metal grade, Romil, UK), with 30 s of MQ water between reagents. Between each analysis a single cleaning cycle was run to return the background to within 1% of the initial background level. Bromine was calibrated by external calibration using standards of 10 to 4,000 pg g^−1^, prepared by diluting a 1,000 mg L^−1^ standard solution (TraceCERT^®^ purity grade, Sigma Aldrich, MO, USA) in MQ water. All calibration curves showed correlation coefficients greater than 0.99 (df = 4, p = 0.05).

The Tropospheric HAlogen chemistry MOdel (THAMO)^43^ is a one dimensional chemistry transport model which includes a multistep implicit-explicit (MIE) integration routine, coupled to a vertical diffusion routine. The model comprises three main components: i) a chemistry module that includes photochemical, gas phase, uptake and surface heterogeneous reactions using the MIE procedure; ii) a transport module that includes vertical eddy diffusion; and, iii) a radiation scheme that calculates the solar irradiance as a function of altitude, wavelength and solar zenith angle (SZA). The model includes 200 boxes at a vertical resolution of 5 m and a total height of 1 km. The lowest level is the surface (ocean, sea ice or landmass) where gas phase deposition and upward flux has been implemented. The chemical scheme implements the so called “bromine explosion”, an autocatalytic cycle of releasing bromine from the ice to the atmosphere^44^. Further information regarding THAMO is included in the Supplementary material.

## Additional Information

**How to cite this article**: Spolaor, A. *et al.* Canadian Arctic sea ice reconstructed from bromine in the Greenland NEEM ice core. *Sci. Rep.* **6**, 33925; doi: 10.1038/srep33925 (2016).

## Supplementary Material

Supplementary Information
