# Supplementary material for: Canadian Arctic sea ice reconstructed from bromine in the Greenland NEEM ice core
Source: Sci Rep. 2016 Sep 21;6:33925. doi: 10.1038/srep33925 (PMC5030631; doi:10.1038/srep33925)
Supplement: Supplementary Information [file srep33925-s1.pdf]

Canadian Arctic sea ice reconstructed from bromine in the Greenland NEEM ice core

Andrea Spolaor[1,2], *Paul Vallelonga[3], Clara Turetta[2], Niccolò Maffezzoli[3], Giulio Cozzi[2], Jacopo Gabrieli[2], Carlo Barbante[1,2], Kumiko Goto-Azuma[4], Alfonso Saiz-Lopez[5], Carlos A. Cuevas[5], Dorthe Dahl-Jensen[3].

1. Ca'Foscari University of Venice, Department of Environmental Science, Informatics and Statistics, Via Torino 155, 30172 Mestre, Venice
2. Institute for the Dynamics of Environmental Processes, IDPA-CNR, Via Torino 155, 30172 Mestre, Venice
3. Centre for Ice and Climate, Niels Bohr Institute, University of Copenhagen, Juliane Maries Vej 30, Copenhagen Ø 2100 Denmark
4. National Institute of Polar Research, 10-3 Midori-cho, Tachikawa Tokyo, 190-8518, Japan
5. Department of Atmospheric Chemistry and Climate, Institute of Physical Chemistry Rocasolano, CSIC, Serrano 119, 28006 Madrid, Spain

1. THAMO model description

The Tropospheric HAlogen chemistry MOdel (THAMO)[1] is a one dimensional chemistry transport model which includes a multistep implicit-explicit (MIE) integration routine[2], coupled to a vertical diffusion routine[3]. The model is also coupled to a dynamic particle production and growth code, and comprises four main components: i) a chemistry module that includes photochemical, gas phase and uptake reactions using the MIE procedure; ii) a transport module that includes vertical eddy diffusion; iii) a radiation scheme that calculates the solar irradiance as a function of altitude, wavelength and solar zenith angle (SZA); and, iv) a particle formation and growth module.

The model includes 200 boxes at a vertical resolution of 5 m and a total height of 1 km. The lowest level is the surface (ocean, sea ice or landmass) where gas phase deposition and upward flux has been implemented. The uptake coefficients for deposition to a specific aerosol surface area have been taken from Sander et al 2006[4] and Atkinson et al 2000[5]. The chemical scheme has been updated according to Mahajan et al 2010[6] implements the so called "bromine explosion", an autocatalytic cycle of releasing

bromine from the snowpack to the atmosphere[7,8]. This cycle starts with the deposition of HOBr and $BrONO_2$ to the snowpack and the reaction with bromide, on the surface of first-year sea ice, to produce active gas phase bromine:

$HOBr + Br^- \rightarrow Br_2 + H_2O$ (S1)

$BrONO_2 + Br^- \rightarrow Br_2 + NO_3^-$ (S2)

The subsequent photolysis of bromine produces BrO, yielding more HOBr and $BrONO_2$ through the reaction with HO_2 and NO_2 respectively:

$BrO + HO_2 \rightarrow HOBr + O_2$ (S3)

$BrO + NO_2 + M \rightarrow BrONO_2 + M$ (S4)

This process leads to an exponential growth of atmospheric gas phase bromine. The autocatalytic cycle continues until the production of HOBr and $BrONO_2$ diminish after ozone is depleted and the deposition of inactive bromine species to the snowpack (HBr and particulate Br^-) prevails. Tables S1, S2 and S3 include all the involved reactions, rates and Henry constants

Table S1. Gas Phase Reactions and Rate Constants used in the THAMO model simulations.

#	Bimolecular Reactions	Rate Constants	References
1.	$O(^1D) + N_2 \rightarrow O + N_2$	$1.8 \times 10^{-11}\ e^{(110/T)}$	9
2.	$O(^1D) + O_2 \rightarrow O + O_2$	$3.2 \times 10^{-11}\ e^{(70/T)}$	9
3.	$O(^1D) + H_2O \rightarrow OH + OH$	2.2×10^{-10}	9
4.	$O(^1D) + CH_4 \rightarrow CH_3 + OH$ (0.75), $CH_3O + H$ (0.2), $HCHO + H_2$ (0.05)	1.5×10^{-10}	9
5.	$O(^1D) + H_2 \rightarrow OH + H$	1.1×10^{-10}	9
6.	$OH + CO \rightarrow H + CO_2$	$1.5 \times 10^{-13} \times (1 + 0.6 \times P_{atm})$	9
7.	$HO_2 + NO \rightarrow NO_2 + OH$	$3.5 \times 10^{-12}\ e^{(250/T)}$	9
8.	$O_3 + HO_2 \rightarrow OH + 2O_2$	$1.1 \times 10^{-14}\ e^{(-500/T)}$	9
9.	$HO_2 + HO_2 \rightarrow H_2O_2 + O_2$	$2.3 \times 10^{-13}\ e^{(600/T)}$	9
10.	$OH + H_2 \rightarrow H_2O + H$	$5.5 \times 10^{-12}\ e^{(-2000/T)}$	9
11.	$O_3 + OH \rightarrow HO_2 + O_2$	$1.6 \times 10^{-12}\ e^{(-940/T)}$	9
12.	$OH + HNO_3 \rightarrow H_2O + NO_3$	$k_0 = 7.2 \times 10^{-15}\ e^{(785/T)}$	9
		$k_2 = 4.1 \times 10^{-16}\ e^{(1440/T)}$	

		$k_3 = 1.9 \times 10^{-33} e^{(725 / T)}$	
		$k = k_0 + (k_3 \times [M] / (1 + k_3 \times [M] / k_2))$	
13.	$H_2O_2 + OH \rightarrow H_2O + HO_2$	$2.9 \times 10^{-12} e^{(-160 / T)}$	[9]
14.	$OH + HO_2NO_2 \rightarrow NO_2 + HO_2 + OH$	$1.3 \times 10^{-12} e^{(380 / T)}$	[9]
15.	$OH + HO_2 \rightarrow H_2O + O_2$	$4.8 \times 10^{-11} e^{(250 / T)}$	[9]
16.	$OH + HONO \rightarrow H_2O + NO_2$	$1.8 \times 10^{-11} e^{(390 / T)}$	[9]
17.	$C_2H_5 + O_2 \rightarrow C_2H_4 + HO_2$	2×10^{-14}	[9],b
18.	$OH + CH_4 \rightarrow CH_3 + H_2O$	$2.45 \times 10^{-12} e^{(-1775 / T)}$	[9]
19.	$O(^3P) + CH_3 \rightarrow CH_3O$	1.1×10^{-10}	[9]
20.	$CH_3O_2 + HO_2 \rightarrow CH_3OOH + O_2$	$3.8 \times 10^{-13} e^{(800 / T)}$	[9]
21.	$CH_3OOH + OH \rightarrow CH_3(O)O + H_2O$	$0.7 \times 3.8 \times 10^{-12} e^{(200 / T)}$	[9]
22.	$CH_3O + O_2 \rightarrow CH_2O + HO_2$	$3.9 \times 10^{-14} e^{(-900 / T)}$	[9]
23.	$OH + HCHO \rightarrow H_2O + HCO$	$8.8 \times 10^{-12} e^{(25 / T)}$	[9]
24.	$HCO + O_2 \rightarrow CO + HO_2$	$3.5 \times 10^{-12} e^{(140 / T)}$	[9]
25.	$CH_3O_2 + CH_3O_2 \rightarrow 2CH_3O + O_2$ 29%	$0.29 \times 2.5 \times 10^{-13} e^{(190 / T)}$	[9]
26.	$NO + CH_3O_2 \rightarrow NO_2 + CH_3O$	$3 \times 10^{-12} e^{(280 / T)}$	[9]
27.	$NO + O_3 \rightarrow NO_2 + O_2$	$2 \times 10^{-12} e^{(-1400 / T)}$	[9]
28.	$NO + NO_3 \rightarrow 2NO_2$	$1.5 \times 10^{-11} e^{(170 / T)}$	[9]
29.	$NO_3 + HCHO \rightarrow$ Products	5.8×10^{-16}	[9],b
30.	$HO_2 + SO_2 \rightarrow$ Products	1×10^{-18}	[9],b
31.	$N_2O_5 + H_2O \rightarrow 2HNO_3$	2.5×10^{-22}	[9],b
32.	$NO_2 + O_3 \rightarrow NO_3 + O_2$	$1.2 \times 10^{-13} e^{(-2450 / T)}$	[9]
33.	$OH + O(^3P) \rightarrow H + O_2$	$2.2 \times 10^{-11} e^{(120 / T)}$	[9]
34.	$O(^3P) + HO_2 \rightarrow OH + O_2$	$3 \times 10^{-11} e^{(200 / T)}$	[9]
35.	$H_2O_2 + O(^3P) \rightarrow OH + HO_2$	$1.4 \times 10^{-12} e^{(-2000 / T)}$	[9]
36.	$OH + OH \rightarrow H_2O + O(^3P)$	$4.2 \times 10^{-12} e^{(-240 / T)}$	[9]
37.	O_3 + Alkenes $\rightarrow$ Products	$1.2 \times 10^{-14} e^{(-2630 / T)}$	[9],b
38.	$NO_3 + CO \rightarrow$ Products	4×10^{-19}	[9],b
39.	$OH + CH_3OOH$ $\rightarrow CH_2OOH + H_2O \rightarrow CH_2O + OH + H_2O$	$0.3 \times 3.8 \times 10^{-12} e^{(200 / T)}$	[9]
40.	$O(^3P) + HCHO \rightarrow OH + HCO$	$3.4 \times 10^{-11} e^{(-1600 / T)}$	[9]
41.	$HCHO + HO_2 \rightarrow HO_2CH_2O$	$6.7 \times 10^{-15} e^{(600 / T)}$	[9]
42.	$H + O_3 \rightarrow OH + O_2$	$1.4 \times 10^{-10} e^{(-470 / T)}$	[9]

43.	$HO_2 + H \rightarrow 2OH$	$0.9 \times 8.1 \times 10^{-11}$	[9]
44.	$O(^3P) + HO_2NO_2 \rightarrow$ Products	$7.8 \times 10^{-11}\, e^{(-3400 / T)}$	[9]
45.	$O(^1D) + O_3 \rightarrow 2O_2$	1.2×10^{-10}	[9]
46.	$O(^1D) + O_3 \rightarrow O_2 + 2O$	1.2×10^{-10}	[9]
47.	$CH_3O_2 + SO_2 \rightarrow$ Products	5×10^{-17}	[10],b
48.	$NO_3 + HO_2 \rightarrow OH + NO_2 + O_2$	3.5×10^{-12}	[9]
49.	$CH_3 + O_3 \rightarrow$ Products	$5.4 \times 10^{-12}\, e^{(-220 / T)}$	[9]
50.	$SO_2 + O_3 \rightarrow SO_3 + O_2$	$3 \times 10^{-12}\, e^{(-7000 / T)}$	[9],b
51.	$NO_3 + OH \rightarrow NO_2 + HO_2$	2.2×10^{-11}	[9]
52.	$O_3 + O(^3P) \rightarrow 2O_2$	$8 \times 10^{-12}\, e^{(-2060 / T)}$	[9]
53.	$O_3 + HONO \rightarrow O_2 + HNO_3$	5×10^{-19}	[9],b
54.	$CH_3O_2 + O_3 \rightarrow$ Products	3×10^{-17}	[9],b
55.	NO_3 + Alkenes $\rightarrow HOCH_2CH_2 + NO_2$	3×10^{-14}	[10]
56.	$SO_2 + NO_2 \rightarrow$ Products	2×10^{-26}	[10],b
57.	NO_3 + Alkanes $\rightarrow C_2H_5 + HNO_3$	3.6×10^{-17}	[10]
58.	$CH_3O_2 + CH_3O_2 \rightarrow CH_2O + CH_3OH + O_2$	$0.71 \times 2.5 \times 10^{-13}\, e^{(190 / T)}$	[9]
59.	$NO_2 + NO_3 \rightarrow NO + NO_2 + O_2$	$4.5 \times 10^{-14}\, e^{(-1260 / T)}$	[9]
60.	OH + Alkanes $\rightarrow C_2H_5 + H_2O$	$1.1 \times 10^{-11} e^{(-1100 / T)}$	[10]
61.	$C_2H_5O_2 + NO \rightarrow NO_2 + C_2H_5O$	$2.6 \times 10^{-12}\, e^{(365 / T)}$	[9]
62.	$CH_3CHO + NO_3 \rightarrow$ $HNO_3 + CH_3CO\ (\rightarrow CH_3C(O)O_2)$	$1.4 \times 10^{-12}\, e^{(-1900 / T)}$	[9]
63.	$CH_3CHO + O(^3P) \rightarrow$ $OH + CH_3CO\ (\rightarrow CH_3C(O)O_2)$	$1.8 \times 10^{-11}\, e^{(-1100 / T)}$	[9]
64.	$CH_3CHO + OH \rightarrow$ $H_2O + CH_3CO\ (\rightarrow CH_3C(O)O_2)$	$5.6 \times 10^{-12}\, e^{(270 / T)}$	[9]
65.	$O(^3P) + H_2 \rightarrow OH + H$	4.11×10^{-18}	[10]
66.	$NO + CH_3C(O)O_2 \rightarrow NO_2 + CH_3 + CO_2$	$5.3 \times 10^{-12}\, e^{(360 / T)}$	[9]
67.	$OH + C_2H_5OOH \rightarrow C_2H_4OOH + H_2O$	3.64×10^{-12}	[10]
68.	$OH + C_2H_5OOH \rightarrow C_2H_5O_2 + H_2O$	5.95×10^{-12}	[10]
69.	$NO_2 + O(^3P) \rightarrow NO + O_2$	$6.5 \times 10^{-12}\, e^{(120 / T)}$	[9]
70.	$NO_3 + O(^3P) \rightarrow NO_2 + O_2$	1×10^{-11}	[9]
71.	$HNO_3 + O(^3P) \rightarrow NO_3 + OH$	3×10^{-17}	[9],b
71.	$C_2H_5O + O_2 \rightarrow CH_3CHO + HO_2$	$6.3 \times 10^{-14}\, e^{(-550 / T)}$	[9]
73.	$HO_2CH_2O \rightarrow HO_2 + CH_2O$	$2.4 \times 10^{12}\, e^{(-7000 / T)}$	[10]

74.	$HO_2CH_2O + HO_2 \rightarrow HCOOH + O_2 + H_2O$	$5.6 \times 10^{-15}\, e^{(2300 / T)}$	[10]

Halogen chemistry

75.	$I + O_3 \rightarrow IO + O_2$	$2 \times 10^{-11}\, e^{(-890 / T)}$	[9]
76.	$I + HO_2 \rightarrow HI + O_2$	$1.5 \times 10^{-11}\, e^{(-1190 / T)}$	[9]
77.	$IO + NO \rightarrow I + NO_2$	$7.3 \times 10^{-12}\, e^{(330 / T)}$	[9]
78.	$IO + HO_2 \rightarrow HOI + O_2$	5.8×10^{-11}	[9]
79.	$IO + IO \rightarrow OIO + I / I_2O_2$	8.6×10^{-11}	[11]
80.	$IO + OIO (+M) \rightarrow I_2O_3$	1.5×10^{-11}	[11]
81.	$IONO_2 \rightarrow IO + NO_2$	$2.07 \times 10^{15}\, e^{(-11859 / T)}$	[9]
82.	$OH + HI \rightarrow I + H_2O$	3×10^{-11}	[9]
83.	$HOI + OH \rightarrow IO + H_2O$	2×10^{-13}	[9]
84.	$IO + DMS \rightarrow$ Products	1.2×10^{-14}	[9]
85.	$INO_2 \rightarrow I + NO_2$	$(2.4 / 0.005) \times 2.07 \times 10^{15}\, e^{(-11859 / T)}$	[9]
86.	$Br + O_3 \rightarrow BrO + O_2$	$1.7 \times 10^{-11} e^{(-800/T)}$	[9]
87.	$HBr + OH \rightarrow Br + H_2O$	1.1×10^{-11}	[9]
88.	$Br + HO_2 \rightarrow HBr + O_2$	$1.5 \times 10^{-11} e^{(-600/T)}$	[9]
89.	$Br + HCHO \rightarrow HBr + HCO$	$7.7 \times 10^{-13}\, e^{(-580/T)}$	[9]
90.	$Br + CH_3CHO \rightarrow HBr\ CH_3CO$	$1.8 \times 10^{-12}\, e^{(-460/T)}$	[9]
91.	$BrO + HO_2 \rightarrow HOBr + O_2$	$3.4 \times 10^{-12} e^{(540/T)}$	[9]
92.	$BrO + NO \rightarrow Br + NO_2$	$8.8 \times 10^{-12} e^{(260/T)}$	[9]
93.	$BrO + CH_3SCH_3 \rightarrow CH_3SOCH_3 + Br$	$1.5 \times 10^{-14} e^{(850/T)}$	[9]
94.	$BrO + BrO \rightarrow 2Br + O_2$	$2.4 \times 10^{-12} e^{(40/T)}$	[9]
95.	$BrO + BrO \rightarrow Br_2 + O_2$	$2.8 \times 10^{-14} e^{(860/T)}$	[9]
96.	$BrONO_2 \rightarrow BrO + NO_2$	$2.8 \times 10^{13}\, e^{-(12360/T)}$	[12]
97.	$BrO + IO \rightarrow Br + I + O_2 / Br + OIO$	$1.5 \times 10^{-12} e^{(510/T)}$	[9]
98.	$Cl + CH_4 \rightarrow HCl + CH_3$	$1.1 \times 10^{-11} e^{(-1400/T)}$	[9]
99.	$HCl + OH \rightarrow H_2O + Cl$	$2.6 \times 10^{-12} e^{(-350/T)}$	[9]
100.	$Cl + O_3 \rightarrow ClO + O_2$	$2.9 \times 10^{-11} e^{(-260/T)}$	[9]
101.	$ClO + HO_2 \rightarrow HOCl + O_2$	$5.0 \times 10^{-12} e^{(700/T)}$	[9]
102.	$ClO + NO \rightarrow Cl + NO_2$	$6.4 \times 10^{-12} e^{(290/T)}$	[9]
103.	$ClO + IO \rightarrow 0.2\,(I + Cl + O_2)$	$1.3 \times 10^{-12} e^{(280/T)}$	[9]
104.	Cl + Alkanes $\rightarrow HCl + CH_3OO$	$5.7 \times 10^{-11} e^{(-90/T)}$	[9, 10]
105.	Cl + Alkenes $\rightarrow HCl + CH_3OO$	1.0×10^{-10}	[9, 10]

106.	$Cl + HCHO \rightarrow HCl + HO_2 + CO$	$7.3 \times 10^{-11}\ e^{(-30/T)}$	9
107.	$ClO + CH_3OO \rightarrow Cl + HCHO + HO_2$	$2.2 \times 10^{-12}\ e^{(-115/T)}$	9
108.	$ClO + ClO \rightarrow Cl_2O_2$	3.5×10^{-13}	9
109.	$Cl_2O_2 \rightarrow ClO + ClO$	50	9
110.	$ClONO_2 \rightarrow ClO + NO_2$	0.0022	9
111.	$Cl + ClONO_2 \rightarrow Cl_2 + NO_3$	$9.6 \times 10^{-12}\ e^{(140/T)}$	9
112.	$Cl + H_2O_2 \rightarrow HCl + HO_2$	$4.1 \times 10^{-13} e^{(-980/T)}$	9
113.	$Br_2 + Cl \rightarrow BrCl + Br$	$1.2 \times 10^{-10} e^{(-260/T)}$	9, 10
114.	$BrCl + Br \rightarrow Br_2 + Cl$	3.3×10^{-15}	9, 10
115.	$Cl_2 + Br \rightarrow BrCl + Cl$	1.1×10^{-15}	9, 10
116.	$BrCl + Cl \rightarrow Cl_2 + Br$	1.5×10^{-11}	9, 10
117.	$ClO + BrO \rightarrow Br + OClO$	$6.0 \times 10^{-12} e^{(550/T)}$	9, 10
118.	$ClO + BrO \rightarrow Br + Cl + O_2$	$5.6 \times 10^{-12} e^{(260/T)}$	9, 10
119.	$ClO + BrO \rightarrow BrCl + O_2$	$1.1 \times 10^{-12} e^{(290/T)}$	9, 10

	**Recombination Reactions**		
1.	$O(^1D) + N_2\ (+M) \rightarrow N_2O\ (+M)$	$[M] \times 3.5 \times 10^{-37} \times (T / 300)^{-0.6}$	9
2.	$HO_2 + HO_2\ (+M) \rightarrow H_2O_2\ (+M)$	$[M] \times 1.7 \times 10^{-33}\ e^{(1000 / T)}$	9
3.	$H + O_2\ (+ M) \rightarrow HO_2\ (+ M)$	$k_0 = 5.7 \times 10^{-32} \times (T / 300)^{-1.6}$	9
		$k_\infty = 7.5 \times 10^{-11}$	
4.	$O_2 + O(^3P) \rightarrow O_3$	$[M] \times 6 \times 10^{-34} \times (T / 300)^{-2.3}$	9
5.	$NO_2 + OH \rightarrow HNO_3$	$k_0 = 2.5 \times 10^{-30} \times (T / 300)^{-4.4}$	9
		$k_\infty = 1.6 \times 10^{-11} \times (T / 300)^{-1.7}$	
6.	$NO + OH\ (+ M) \rightarrow HONO\ (+ M)$	$k_0 = 7 \times 10^{-31} \times (T / 300)^{-2.6}$	9
		$k_\infty = 1.5 \times 10^{-11} \times (T / 300)^{-0.5}$	
7.	$HO_2 + NO_2\ (+ M) \rightarrow HO_2NO_2\ (+ M)$	$k_0 = 1.8 \times 10^{-31} \times (T / 300)^{-3.2}$	9
		$k_\infty = 4.7 \times 10^{-12} \times (T / 300)^{-1.4}$	
8.	$HO_2NO_2 \rightarrow HO_2 + NO_2$	$k_R = k_F / k_{EQ}$	9
		$k_R = k_F / (2.1 \times 10^{-27}\ e^{(10900 / T)})$	
9.	$O_2 + CH_3\ (+ M) \rightarrow CH_3O_2\ (+ M)$	$k_0 = 4.5 \times 10^{-31} \times (T / 300)^{-3}$	9
		$k_\infty = 1.8 \times 10^{-12} \times (T / 300)^{-1.7}$	
10.	$NO_2 + NO_3\ (+ M) \rightarrow N_2O_5\ (+ M)$	$k_0 = 2.2 \times 10^{-30} \times (T / 300)^{-3.9}$	9
		$k_\infty = 1.5 \times 10^{-12} \times (T / 300)^{-0.7}$	

11.	N_2O_5 (+ N_2) → NO_2 + NO_3 (+ N_2)	$k_R = k_F / k_{EQ}$	9
		$k_R = k_F / (2.7 \times 10^{-27} e^{(11000 / T)})$	
12.	OH + OH (+ M) → H_2O_2 (+ M)	$k_0 = 6.2 \times 10^{-31} \times (T / 300)^{-1}$	9
		$k_\infty = 2.6 \times 10^{-11}$	
13.	NO + $O(^3P)$ (+ M) → NO_2 (+ M)	$k_0 = 9 \times 10^{-32} \times (T / 300)^{-1.5}$	9
		$k_\infty = 3 \times 10^{-11}$	
14.	NO_2 + $O(^3P)$ (+ M) → NO_3 (+ M)	$k_0 = 9 \times 10^{-32} \times (T / 300)^{-2}$	9
		$k_\infty = 2.2 \times 10^{-11}$	
15.	SO_2 + OH (+ M) → $HOSO_2$ (+ M)	$k_0 = 3 \times 10^{-31} \times (T / 300)^{-3.3}$	9
		$k_\infty = 1.5 \times 10^{-12}$	
16.	$CH_3C(O)O_2$ + NO_2 (+ M) → PAN (+ M)	$k_0 = 9.7 \times 10^{-29} \times (T / 300)^{-5.6}$	9
		$k_\infty = 9.3 \times 10^{-12} \times (T / 300)^{-1.5}$	
17.	PAN (+ M) → $CH_3C(O)O_2$ + NO_2 (+ M)	$k_R = k_F / k_{EQ}$	9
		$k_R = k_F / (9 \times 10^{-29} e^{(14000 / T)})$	
18.	OH + Alkenes (+ M) → $HOCH_2CH_2$ (+ M)	$k_0 = 1.5 \times 10^{-28} \times (T / 300)^{-0.8}$	9, 10
		$k_\infty = 8.8 \times 10^{-12}$	
19.	C_2H_5 + O_2 (+ M) → $C_2H_5O_2$ (+ M)	$k_0 = 1.5 \times 10^{-28} \times (T / 300)^{-3.8}$	9
		$k_\infty = 8 \times 10^{-12}$	
20.	NO_2 + CH_3O_2 (+ M) → $CH_3O_2NO_2$ (+ M)	$k_0 = 1.5 \times 10^{-30} \times (T / 300)^{-4}$	9
		$k_\infty = 6.5 \times 10^{-12} \times (T / 300)^{-2}$	
21.	$CH_3O_2NO_2$ → CH_3O_2 + NO_2	$k_R= k_F / k_{EQ}$	9
		$k_R = k_F / (1.3 \times 10^{-28} e^{(11200 / T)})$	
22.	I + NO_2 (+ M) → INO_2 (+ M)	$k_0 = 3 \times 10^{-31} \times (T / 300)^{-1}$	9
		$k_\infty = 6.6 \times 10^{-11}$	
		$F_c = e^{(-T / 650)} + e^{(-2600 / T)}$	
23.	IO + NO_2 (+ M) → $IONO_2$ (+ M)	$k_0 = 7.7 \times 10^{-31} \times (T / 300)^{-5}$	9
		$k_\infty = 1.6 \times 10^{-11}$	
		$F_c = 0.4$	
24.	Br + NO_2 + M → $BrNO_2$	$k_0 = 4.2 \times 10^{-31} \times (T / 300)^{-2.4}$	9
		$k_\infty = 2.7 \times 10^{-11} \times (T / 300)^{-0}$	
25.	BrO + NO_2 + M → $BrONO_2$	$k_0 = 5.2 \times 10^{-31} \times (T / 300)^{-3.2}$	9
		$k_\infty = 6.9 \times 10^{-12} \times (T / 300)^{-2.9}$	
25.	ClO + NO_2 + M → $ClONO_2$	$k_0 = 1.6.2 \times 10^{-31} \times (T / 300)^{-3.4}$	9

$k_\infty = 1.5 \times 10^{-11}$

	Photochemical Reactions	References
J1.	$O_3 + h\nu \rightarrow O_2 + O(^1D)$	[9], [10],c
J2.	$H_2O_2 + h\nu \rightarrow 2OH$	[9], [10],c
J3.	$HNO_3 + h\nu \rightarrow OH + NO_2$	[9], [10],c
J4.	$HO_2NO_2 + h\nu \rightarrow OH + NO_3$	[9], [10],c
J5.	$HONO + h\nu \rightarrow OH + NO$	[9], [10],c
J6.	$CH_3OOH + h\nu \rightarrow CH_3O + OH$	[9], [10],c
J7.	$CH_2O + h\nu \rightarrow HCO + H$	[9], [10],c
J8.	$CH_2O + h\nu \rightarrow CO + H_2$	[9], [10],c
J9.	$NO_2 + h\nu \rightarrow NO + O$	[9], [10],c
J10.	$NO_3 + h\nu \rightarrow NO_2 + O$	[9], [10],c
J11.	$N_2O_5 + h\nu \rightarrow NO_2 + NO_3$	[9], [10],c
J12.	$C_2H_5O_2H + h\nu \rightarrow OH + C_2H_5O$	[9], [10],c
J13.	$CH_3CHO + h\nu \rightarrow CH_3 + HCO$	[9], [10],c
J15.	PAN (CH3C(O)O_2NO_2) + $h\nu \rightarrow$ $CH_3C(O)O_2 + NO_2$	[9], [10],c
J16.	$NO_3 + h\nu \rightarrow NO + O_2$	[9], [10],c
J17.	$CH_3I + h\nu \rightarrow CH_3 + I$	[9], [10],c
J18.	$CH_2I_2 + h\nu \rightarrow CH_2I + I \rightarrow CH_2 + 2I$	[9], [10],c
J19.	$CH_2IBr + h\nu \rightarrow CH_2Br + I$	[9], [10],c
J20.	$I_2 + h\nu \rightarrow 2I$	[9], [10],c
J21.	$INO_2 + h\nu \rightarrow I + NO_2$ / $IO + NO$	[9], [10],c
J22.	$IO + h\nu \rightarrow I + O$	[9], [10],c
J23.	$OIO + h\nu \rightarrow I + O_2$	[9], [10],c
J24.	$IONO_2 + h\nu \rightarrow I + NO_3$	[9], [10],c

J25.	$HOI + h\nu \rightarrow I + OH$	[9], [10],c
J26.	$BrO + h\nu \rightarrow Br + O$	[9], [10],c
J27.	$Br_2 + h\nu \rightarrow 2Br$	[9], [10],c
J28.	$IBr + h\nu \rightarrow Br + I$	[9], [10],c
J29.	$BrCl + h\nu \rightarrow Br + Cl$	[9], [10],c
J30.	$BrNO_2 + h\nu \rightarrow Br + NO_2$	[9], [10],c
J31.	$BrONO_2 + h\nu \rightarrow$ 0.7 ($BrO + NO_2$) / 0.3 ($Br + NO_3$)	[9], [10],c
J32.	$HOBr + h\nu \rightarrow Br + OH$	[9], [10],c
J33.	$Cl_2 + h\nu \rightarrow 2Cl$	[9], [10],c
J34.	$ICl + h\nu \rightarrow I + Cl$	[9], [10],c
J35.	$ClO + h\nu \rightarrow Cl + O$	[9], [10],c
J36.	$HOCl + h\nu \rightarrow Cl + OH$	[9], [10],c
J37.	$ClNO_2 + h\nu \rightarrow Cl + NO_2$	[9], [10],c
J38.	$ClONO_2 + h\nu \rightarrow$ 0.9 ($Cl + NO_3$) / 0.1 ($ClO + NO_2$)	[9], [10],c

**Species**	**Deposition velocities, cm s^{-1}**	**References**
HOI	1.0	[13]
HOBr	1.0	d
HOCl	1.0	d
HBr	2.0	d
HCl	2.0	d
HI	1.0	[13]
$BrONO_2$	1.0	d
$IONO_2$	1.0	[13]
$ClONO_2$	1.0	d
INO_2	1.0	d

[a]Units: unimolecular reactions, s^{-1}; photolysis rate constants, s^{-1}; bimolecular reactions, cm^3 $molecule^{-1}$ s^{-1}; termolecular reactions, cm^6 $molecule^{-2}$ s^{-1}, calculated using the formalism of *Sander et al.* (2006)[9], where $k = ((k_o [M]/(1 + k_o[M]/k_\infty)) \times F_c^{\,n}), F_c = 0.6$ (unless otherwise noted) and $n = (1 + (\log_{10}(k_o[M]/k_\infty))^2)^{-1}$.
[b]set as upper limit.
[c]absorption cross-sections taken from *Atkinson et al.*, 2000[10].
[d]deposition velocities estimated.

Table S2. QLL Reactions and Rate Constants used in the THAMO model simulations.

#	Reactions	Rate Constants	References
1.	$HOI + I^- + H^+ \rightarrow I_2 + H_2O$	4.4×10^{12} M^{-2} s^{-1}/(volumetric)^2	14
2.	$I_2 + H_2O \rightarrow HOI + I^- + H^+$	0 s^{-1}	
3.	$HOI + Br^- + H^+ \rightarrow IBr + H_2O$	3.3×10^{12} M^{-2} s^{-1}/(volumetric)^2	15
4.	$IBr + H_2O \rightarrow HOI + Br^- + H^+$	8.0×10^5 s^{-1}	15
5.	$HOI + Cl^- + H^+ \rightarrow ICl + H_2O$	2.9×10^{10} M^{-2} s^{-1}/(volumetric)^2	16
6.	$ICl + H_2O \rightarrow HOI + Cl^- + H^+$	2.4×10^6 s^{-1}	16
7.	$HOBr + Br^- + H^+ \rightarrow Br_2 + H_2O$	1.6×10^{10} M^{-2} s^{-1}/(volumetric)^2	17
8.	$Br_2 + H_2O \rightarrow HOBr + Br^- + H^+$	9.7×10^1 s^{-1}	17
9.	$HOBr + Cl^- + H^+ \rightarrow BrCl + H_2O$	5.6×10^9 M^{-2} s^{-1}/(volumetric)^2	18
10.	$BrCl + H_2O \rightarrow HOBr + Cl^- + H^+$	1.0×10^5 s^{-1}	18
11.	$BrCl + Br^- \rightarrow Br_2Cl^-$	5.0×10^9 M^{-1} s^{-1}/(volumetric)	18
12.	$Br_2Cl^- \rightarrow BrCl + Br^-$	2.8×10^5 s^{-1}	18
13.	$Br_2Cl^- \rightarrow Br_2 + Cl^-$	3.8×10^9 s^{-1}	18
14.	$Br_2 + Cl^- \rightarrow Br_2Cl^-$	5.0×10^9 M^{-1} s^{-1}/(volumetric)	18
15.	$BrCl + Cl^- \rightarrow BrCl_2^-$	5.0×10^9 M^{-1} s^{-1}/(volumetric)	19
16.	$BrCl_2^- \rightarrow BrCl + Cl^-$	1.3×10^9 s^{-1}	19
17.	$HOBr + I^- \rightarrow IBr + OH^-$	5.0×10^9 M^{-1} s^{-1}/(volumetric)	20
18.	$HOCl + Cl^- + H^+ \rightarrow Cl_2 + H_2O$	2.2×10^4 $e^{(-3508/T)}$ M^{-2} s^{-1}/(volumetric)^2	21
19.	$Cl_2 + H_2O \rightarrow HOCl + Cl^- + H^+$	2.2×10^1 $e^{(-8012/T)}$ s^{-1}	21

20.	$HOCl + Br^- + H^+ \rightarrow BrCl + H_2O$	3.5×10^{11} M^{-2} s^{-1}/(volumetric)^2	22
21.	$BrCl + H_2O \rightarrow HOCl + Br^- + H^+$	0 s^{-1}	
22.	$HOCl + I^- + H^+ \rightarrow ICl + H_2O$	3.9×10^{-14} $e^{(-900 / T)}$ M^{-2} s^{-1}/(volumetric)^2	23
23.	$ICl + H_2O \rightarrow HOCl + I^- + H^+$	0 s^{-1}	

Table S3. Henry Constants used in the THAMO model simulations.

Species	Henry Constants	References
IO	4.5×10^2 $e^{(5862(1/T - 1/To))}$ M atm^{-1}	24
HOI	4.5×10^2 $e^{(5862(1/T - 1/To))}$ M atm^{-1}	24, 25
I_2	3.0×10^0 $e^{(4431(1/T - 1/To))}$ M atm^{-1}	26
ICl	1.1×10^2 $e^{(5600(1/T - 1/To))}$ M atm^{-1}	27
IBr	2.4×10^1 $e^{(5600(1/T - 1/To))}$ M atm^{-1}	28
HOBr	9.3×10^1 $e^{(5862(1/T - 1/To))}$ M atm^{-1}	24, 28
Br_2	7.6×10^{-1} $e^{(4094(1/T - 1/To))}$ M atm^{-1}	29
BrCl	9.4×10^{-1} $e^{(5600(1/T - 1/To))}$ M atm^{-1}	25
HOCl	6.7×10^2 $e^{(5862(1/T - 1/To))}$ M atm^{-1}	25
Cl_2	9.1 x 10-^{2} $e^{(2500(1/T - 1/To))}$ M atm^{-1}	30

2. Modelling experiments and results

In this work, THAMO is configured to simulate an air mass moving during five days from the land/ocean, passing over coastal sea ice, to the location of the NEEM ice core in Greenland (Figure S1). Several sensitivity runs were conducted to simulate scenarios with and without first-year sea ice along the trajectory of the air mass.

As the airmass moves, THAMO implements *i*) the release of bromine precursors from the ocean (Br_{2Flux} ~ 1.5×10^9 molecules cm^{-2} s^{-1} depending on actinic flux, and therefore describing a Gaussian profile peaking at midday), *ii*) the surface recycling of bromine in

the first-year sea ice, (according to bromine explosion cycle, reaction equations S1-S4) and *iii*) calculates the deposition of bromine at the NEEM location over the Greenland snowpack. Note that once the airmass reaches Greenland the "bromine explosion" ceases as the bromide content of snowpack over multiyear sea ice is negligible. The model is constrained with typical polar boundary layer mixing ratios: [O_3] = 3 ppbv; [DMS] = 80 pptv; [SO_2] = 15 pptv, [CH_4] = 1,750 ppbv; [CH_3CHO] = 150 pptv; [HCHO] = 150 pptv; [isoprene] = 60 pptv; [propane] = 25 pptv; [propene] = 15 pptv[31,32].

The model is run in three different scenarios; according to the time the airmass spends over ocean, first-year sea ice (with active surface recycling) and Greenland (with no surface recycling):

- Experiment 1: 24 hour over ocean, 24 hour over first-year sea ice and 72 hours over Greenland (Figure 2a).
- Experiment 2: 24 hour over ocean, 48 hour over first-year sea ice and 48 hours over Greenland (Figure 2b).
- Experiment 3: 24 hour over ocean and 96 hours over Greenland land ice (Figure 2c).

Figure 2 shows diurnal averages of the calculated bromine deposition and total gas phase bromine, together with the BrO concentration in the three different scenarios. Blue, grey and yellow shaded zones correspond to ocean, first-year sea ice and Greenland regions, respectively. The model is run to reach steady-state over the ocean, then as the airmass moves the presence of first-year sea ice enhances the amount of bromine in the air mass, increasing almost up to four times when the airmass spends 2 days over the sea ice. Therefore the eventual amount of bromine during the fifth day of simulation clearly depends on the extension of active (first-year) sea ice that the airmass encounters, e.g. on the time the air mass spends recycling bromine over the (first-year) sea ice surface. We simulate that 3, 15 and 30 pptv of total gas phase bromine are produced at the end of the simulation for the scenarios with 0 h, 24 h and 48 h over first-year sea ice, respectively. Finally, the deposition of bromine to ice during the last day of simulation is one order of magnitude higher in the scenario with two days of first-year sea ice, compared to that without first-year sea ice. Overall, the results show that the presence, and extent, of first-year sea ice on the coast of Greenland can have a

significant effect on the levels of bromine that are transported and deposited at the location of the NEEM ice core.

3. Arctic first-year sea ice

Based on the evidence for a dominant westerly wind circulation pattern over northern Greenland described above, we consider the main regions of Arctic FYSI which could act as a source for the bromine enrichment observed at NEEM. In the following discussion we refer to the Arctic locations described in Figure S2. It is only during the satellite period, since 1979, that detailed observations of daily sea ice extent are available[33]. We compare two satellite observations showing an Arctic sea ice maximum (March 2015) and a following Arctic sea ice minimum (September 2015) to give an example of the regions where FYSI and MYSI are present in the Arctic (Fig S3). From the September sea ice minimum, it is clear that MYSI is centred on the central Arctic Ocean around the north pole and extending down to the north coast of Greenland and Canadian archipelago.

FYSI develops in the sectors of the Arctic Ocean that are not covered by MYSI. These can be primarily divided into three zonal regions: The Canadian Arctic, Hudson Bay and Baffin Bay; the Beaufort, Chukchi, East Siberian and Laptev seas; and the Barents and Kara seas. Of these, the Canadian Arctic, Baffin and Hudson bays are the FYSI areas located closest to, and directly upwind of, the NEEM ice core site. 10-day airmass back trajectories display more distant air mass origins corresponding to source regions in the Beaufort and Chukchi seas, and possibly also a minor contribution from the Sea of Okhotsk. These more distant regions cannot be discounted as a contributing, but minor, source of bromine enrichment.

4. Sources of sea salt and mineral aerosols deposited at NEEM

The source of aerosols arriving at the NEEM ice core site have been established primarily by back-trajectory analysis. There is widespread agreement between several studies, of a westerly circulation system and airmasses sourced from northern North America (indicated by biomass burning proxies ammonium, black carbon and levoglucosan) and central Asia (indicated by dust geochemistry and provenance

studies). Gfeller et al.[34] describe a 30-year data set of 10-day airmass back-trajectories showing "that most of the air parcels arriving at NEEM are originating over northern North America and follow the Greenland coast to NEEM". A brief review of biomass burning markers in the NEEM ice core (Zennaro et al., 2014[35]) finds varying levels of apportionment between northern North America (40 to 55%) and east Asia (20 to 40%) among three different studies of backtrajectory data. Most recently, Kang et al.[36] compiled daily 10-day airmass back-trajectories for the period September 2005-August 2006. They found airmasses originated from various directions (including the Canadian Arctic, North America, and the North Atlantic) although "the major contributor to Greenland aerosols was an air mass passing over the Canadian Arctic and North America during the winter-spring seasons.".

Considering that our enrichment model considers only a 5 day airmass history, we expect that sea salt aerosol will be incorporated into the dominant aerosol transport pattern as it travels toward the NEEM site. Such an interpretation is consistent with our apportionment of Baffin Bay and the Canadian sector of the Arctic Ocean as the likely sources of bromine enrichment observed at NEEM. Recent observational evidence of a bromine explosion event in the Canadian high Arctic (Zhao et al., 2015[37]) confirms a westerly circulation pattern and transport toward northwest Greenland.

5. NEEM ice core sampling resolution

Samples were obtained from the Continuous Flow Analysis (CFA) system operated at the NEEM drilling site. One discrete sample was collected for each 1.10 m of melted ice, although not all discrete samples were analysed for Br and Na. Two considerations are necessary when interpreting the sample resolution: the frequency of sampling; and the number of years covered in each sample. Regarding the frequency of sampling, relatively few samples cover the Holocene (82 samples covering 1,411 m of ice and 10.5 ky) although there are more for the glacial (247 samples covering 800 m of ice and 110 ky). The samples are not evenly distributed, with 62 samples covering the past 3 ky and only 20 samples for the early Holocene. The densest sampling covered the period 25 to 50 ky ago (Figure 3b).

An additional consideration is the number of years integrated in each sample: for the late Holocene, each sample integrates only 4 to 6 years of deposition. For the early

Holocene, each sample integrates approximately 20 years, and for the glacial each samople integrates approximately 60 (“warm” glacial interstadial) to 120 (“cold” glacial stadial) years. This is due to the maintenance of a fixed sampling interval (1.10 m) over the length of the core, despite the thinning of each annual layer with depth.

6. Interpretation and quantification of Bromine enrichment in the NEEM ice core

It is important to note that the absence of bromine recycling should not be exclusively interpreted as a lack of FYSI. Analogous to the careful interpretation required for sea ice diatom products (e.g. IP_{25})[38], the absence of Br enrichment can also occur in the absence of all forms of sea ice, that is, the presence of open ocean conditions. In this sense, the relation of Br enrichment to FYSI is multivariate and all available data must be taken into account when interpreting the data. In the case of the NEEM data presented here, we employ the $\delta^{18}O$ paleotemperature proxy from the NGRIP ice core to evaluate overall temperature conditions. During the glacial period, when Arctic temperatures were at least 20 ºC colder than the present[39], it is unlikely that open ocean conditions existed within the Arctic Ocean. Hence, the absence of Br enrichment has been interpreted as an absence of FYSI rather than a presence of open waters in the Arctic Ocean.

The increased Br enrichment values observed during glacial interstadials and the Holocene are interpreted as a progressive increase in the proportion of FYSI coverage in the area sampled by airmasses arriving at the NEEM site. Consequently, the “maximum FYSI” case should also be considered: that the Br enrichment signal might be “saturated”. In this sense, “saturation” corresponds to a scenario in which the airmass sampling area is completely occupied by FYSI, but FYSI continues to extend beyond the area sampled. We evaluate the immediate sampling area of the NEEM site to include the Hudson and Baffin bays and the Canadian Archipelago. Of these, only Baffin Bay is open-sided, that is, available to unconstrained growth (Figure S4). The presence of MYSI in the Arctic Ocean under current climate conditions suggests that Br enrichment in the NEEM ice core is not currently “saturated” and that Br enrichment values may be expected to increase as MYSI sea ice extent is expected to decrease in future. Although there is a substantial data gap in the NEEM data corresponding to the

Holocene climate period, we have used the NEEM Br enrichment data and observed FYSI extent areas to produce a quantitative evaluation of FYSI extent in the Canadian Arctic during the early Holocene. Based on the backtrajectory studies described in the main text, we consider the Canadian Arctic to be the primary source of aerosols entrained and transported to the NEEM site. This aerosol entrainment area comprises the regions of Hudson Bay, Baffin Bay and the Canadian Archipelago (Figures S2 and S4). We assume that sea ice variability observed during the satellite era (1979-2013) can be considered representative of the late Holocene, due to the findings of Kinnard et al. [40], indicating that the range of sea ice variability during the last 1,500 years was within the range observed by satellite. We note that the bromine enrichment values available for the early Holocene do not cover the warmest period of the Holocene Climate Optimum, and hence we consider our evaluation of 10^6 km^2 FYSI increase during the early Holocene to be a conservative estimate.

Figure S1: Schematic of chemical and physical processes in the THAMO chemical transport model scenarios. Bromine enrichment of an air mass occurs through heterogeneous recycling in first year sea ice (bromine explosion) which is then transported over the Greenland ice sheet.

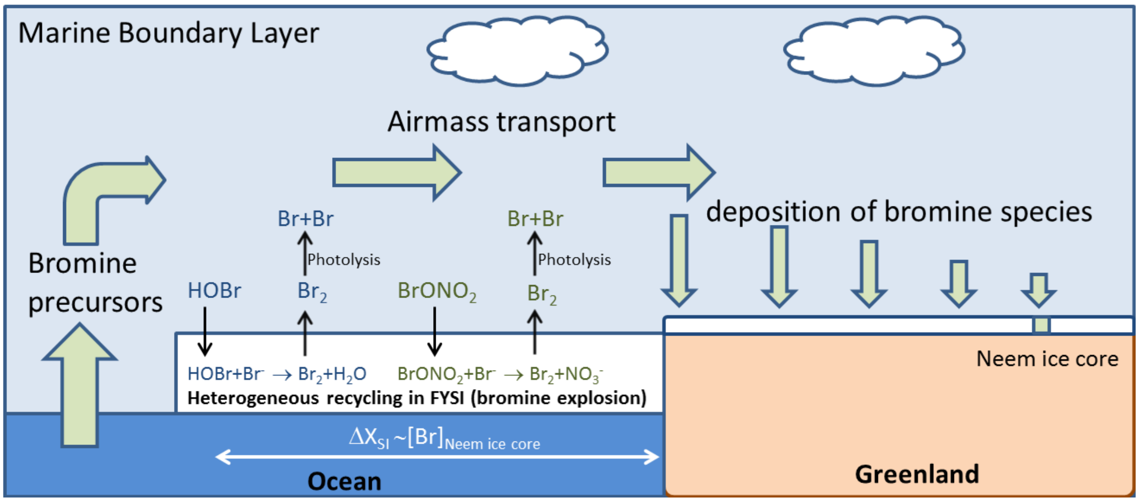

Figure S2. Map of the Arctic Ocean with regional location names. Representative locations corresponding to the Arctic seas described in the text are shown here. Image from NSIDC[33].

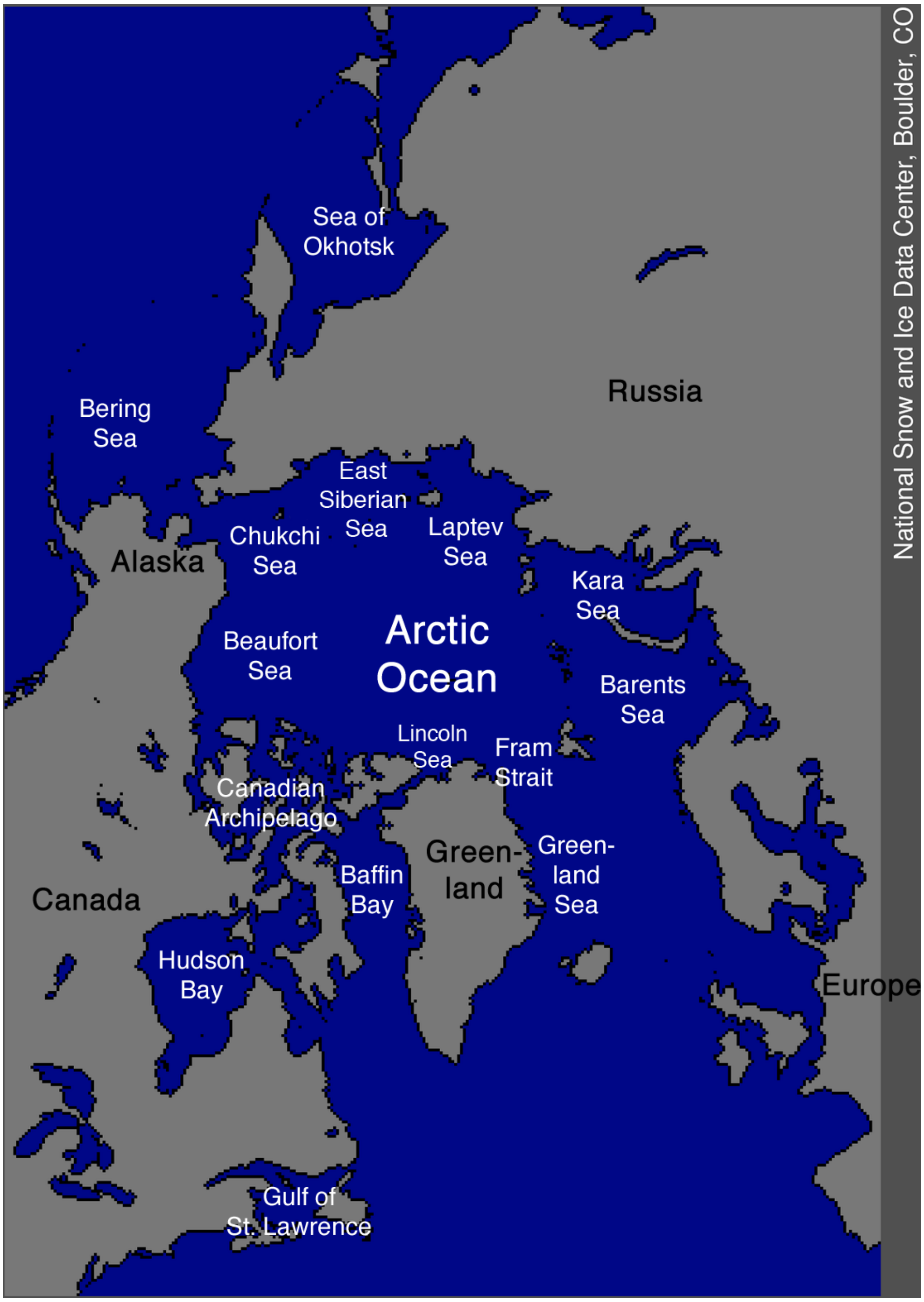

Figure S3. Minimum and maximum Arctic sea ice extent in 2015. Subsequent Arctic sea ice maxima (left panel) and minima (right panel) are shown as an example of the regions where first year sea ice is present in the Arctic. The magenta line shows the median (1981-2010) location of the sea ice edge for each month. It is apparent that large areas of FYSI occur along the northern coasts of Canada, Alaska and the CIS. Image from NSIDC[33].

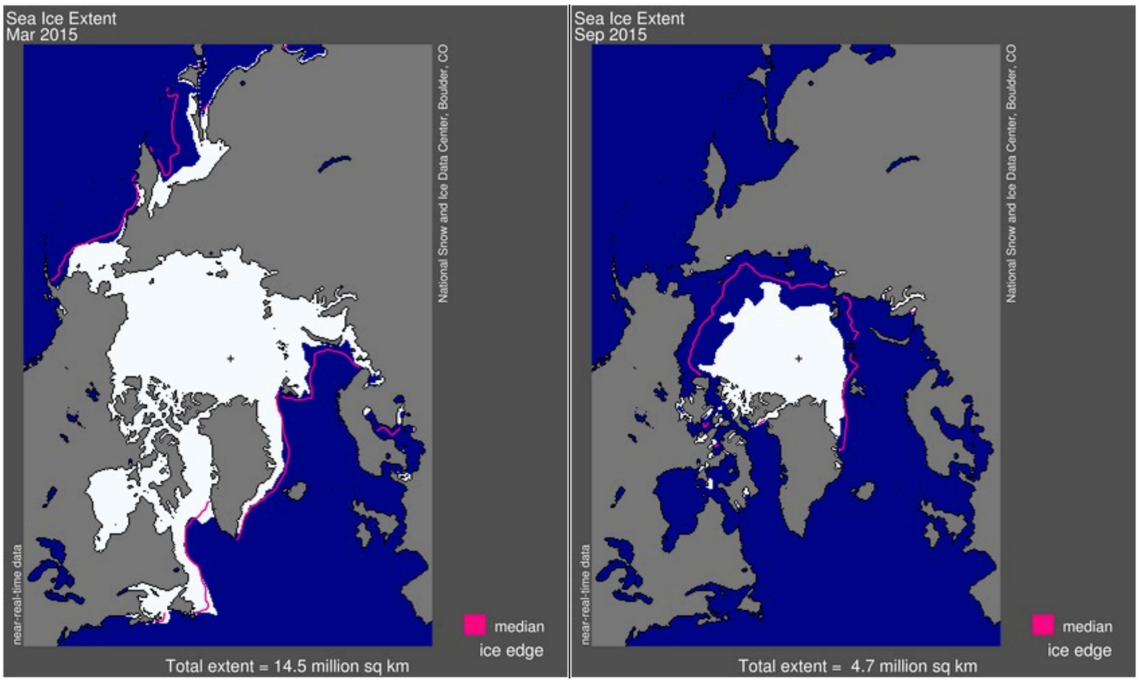

Figure S4. Polygons used to calculate sea ice parameters from satellite observations. The coloured shapes in the figure correspond to various regions named in Figure S2. It can be seen that most of the regions within the Arctic Ocean are constrained by landmasses, whereas Baffin Bay, Greenland Sea and Bering Sea (among others) are unconstrained. Image from NSIDC[33].

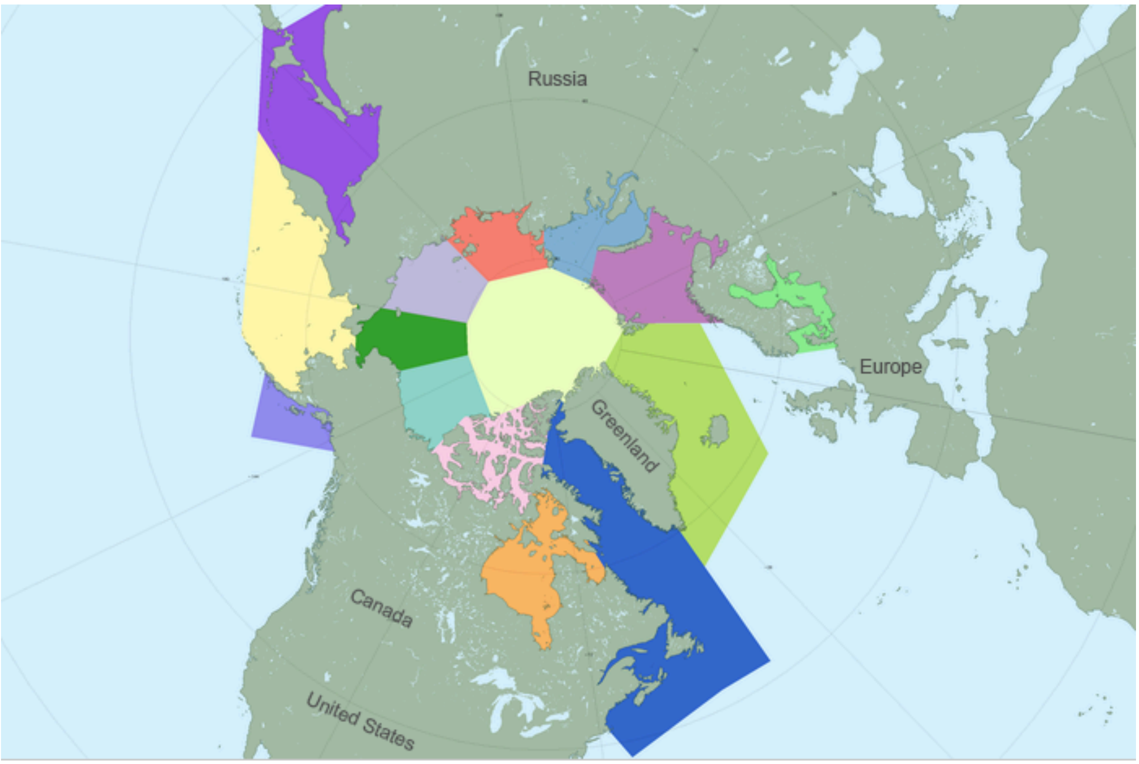

References

1 Saiz-Lopez, A. *et al.* On the vertical distribution of boundary layer halogens over coastal Antarctica: implications for O_3, HO_x, NO_x and the Hg lifetime. *Atmos. Chem. Phys.* **8**, 887-900 (2008).

2 Jacobson, M. Z. *Fundamentals of atmospheric modelling*. 2nd edn, (Cambridge Univ. Press, 2005).

3 Shimazaki, T. *Minor constituents in the middle atmosphere*. (D. Reidel, 1985).

4 Sander, R., Burrows, J. P. & Kaleschke, L. Carbonate precipitation in brine – a potential trigger for tropospheric ozone depletion events. *Atmos. Chem. Phys.* **6**, 4653-4658 (2006).

5 Atkinson, R. *et al.* Evaluated kinetic and photochemical data for atmospheric chemistry: Supplement VIII. Halogen species – IUPAC subcommittee on gas kinetic data evaluation for atmospheric chemistry *J. Phys. Chem. Ref. Data* **29**, 167-266 (2000).

6 Mahajan, A. S. *et al.* Measurement and modelling of tropospheric reactive halogen species over the tropical Atlantic Ocean. *Atmos. Chem. Phys.* **10**, 4611-4624 (2010).

7 Wennberg, P. Atmospheric chemistry: Bromine explosion. *Nature* **397**, 299-301 (1999).

8 Toyota, K. *et al.* Analysis of reactive bromine production and ozone depletion in the Arctic boundary layer using 3-D simulations with GEM-AQ: inference from synoptic-scale patterns. *Atmos. Chem. Phys.* **11**, 3949-3979 (2011).

9 Sander, S. P. *et al.* Chemical kinetics and photochemical data for use in atmospheric studies. Evaluation Number 15. (JPL-NASA, Pasadena, 2006).

10 Atkinson, R. *et al.* Evaluated Kinetic and Photochemical Data for Atmospheric Chemistry: Supplement VIII, Halogen Species Evaluation for Atmospheric Chemistry. *J. Phys. Chem. Ref. Data* **29**, 167 (2000).

11 Martín, J. C. G., Spietz, P. & Burrows, J. P. Spectroscopic studies of the I2/O3 photochemistry: Part 1: Determination of the absolute absorption cross sections of iodine oxides of atmospheric relevance. *J. Photochemistry and Photobiology A.* **176**, 15-38 (2005).

12 Orlando, J. J. & Tyndall, G. S. Rate Coefficients for the Thermal Decomposition of BrONO2 and the Heat of Formation of BrONO2. *J. Phys. Chem.* **100**, 19398-19405 (1996).

13 Jenkin, M. E. Technical Report AEA-EE-0405. (Atomic Energy Authority, Harwell Laboratory, Oxfordshire, UK, 1992).

14 Eigen, M. & Kustin, K. The Kinetics of Halogen Hydrolysis. *J. Am. Chem. Soc.* **84**, 1355-1361 (1962).

15 Troy, R. C., Kelley, M. D., Nagy, J. C. & Margerum, D. W. Non-metal redox kinetics: iodine monobromide reaction with iodide ion and the hydrolysis of IBr. *Inorg. Chem.* **30**, 4838-4845 (1991).

16 Wang, Y. L., Nagy, J. C. & Margerum, D. W. Kinetics of hydrolysis of iodine monochloride measured by the pulsed-accelerated-flow method. *J. Am. Chem. Soc.* **111**, 7838-7844 (1989).

17 Beckwith, R. C., Wang, T. X. & Margerum, D. W. Equilibrium and Kinetics of Bromine Hydrolysis. *Inorg. Chem.* **35**, 995-1000 (1996).

18 Wang, T. X., Kelley, M. D., Cooper, J. N., Beckwith, R. C. & Margerum, D. W. Equilibrium, Kinetic, and UV-Spectral Characteristics of Aqueous Bromine Chloride, Bromine, and Chlorine Species. *Inorg. Chem.* **33**, 5872-5878 (1994).

19 Liu, Q. & Margerum, D. W. Equilibrium and Kinetics of Bromine Chloride Hydrolysis. *Environ. Sci. Technol.* **35**, 1127-1133 (2001).

20 Troy, R. C. & Margerum, D. W. Non-metal redox kinetics: hypobromite and hypobromous acid reactions with iodide and with sulfite and the hydrolysis of bromosulfate. *Inorg. Chem.* **30**, 3538-3543 (1991).
21 Wang, T. X. & Margerum, D. W. Kinetics of Reversible Chlorine Hydrolysis: Temperature Dependence and General-Acid/Base-Assisted Mechanisms. *Inorg. Chem.* **33**, 1050-1055 (1994).
22 Kumar, K. & Margerum, D. W. Kinetics and mechanism of general-acid-assisted oxidation of bromide by hypochlorite and hypochlorous acid. *Inorg. Chem.* **26**, 2706-2711 (1987).
23 Nagy, J. C., Kumar, K. & Margerum, D. W. Non-metal Redox Kinetics - Oxidation of iodide by hypochlorous acid and by nitrogen trichloride measured by the pulsed-accelerated-flow method. *Inorg. Chem.* **27**, 2773-2780 (1988).
24 Huthwelker, T. *et al.* Solubility of HOCl in water and aqueous H2SO4 to stratospheric temperatures. *J. Atmos. Chem.* **21**, 81-95 (1995).
25 Chatfield, R. B. & Crutzen, P. J. Are there interactions of iodine and sulfur species in marine air photochemistry? *J. Geophys. Res.* **95**, 22319–22341 (1990).
26 Palmer, D. A., Ramette, R. W. & Mesmer, R. E. The hydrolysis of iodine: Equilibria at high temperatures. *J. Nucl. Mater.* **130**, 280-286 (1985).
27 Bartlett, W. P. & Margerum, D. W. Temperature Dependencies of the Henry's Law Constant and the Aqueous Phase Dissociation Constant of Bromine Chloride. *Environ. Sci. Technol.* **33**, 3410-3414 (1999).
28 Vogt, R., Crutzen, P. J. & Sander, R. A mechanism for halogen release from sea-salt aerosol in the remote marine boundary layer. *Nature* **383**, 327-330 (1996).
29 Dean, J. A. *Lange's Handbook of Chemistry*. 12 edn, (McGraw-Hill, Inc., 1992).
30 Wilhelm, E., Battino, R. & Wilcock, R. J. Low-pressure solubility of gases in liquid water. *Chem. Rev.* **77**, 219-262 (1977).
31 Read, K. A., Lewis, A. C., Salmon, R. A., Jones, A. E. & Bauguitte, S. OH and halogen atom influence on the variability of non-methane hydrocarbons in the Antarctic Boundary Layer. *Tellus B* **59**, 22-38 (2007).
32 Jones, A. E. *et al.* Chemistry of the Antarctic Boundary Layer and the Interface with Snow: an overview of the CHABLIS campaign. *Atmos. Chem. Phys.* **8**, 3789-3803 (2008).
33 Fetterer, F., Knowles, K., Meier, W. & Savoie, M. Sea Ice Index, 2009-2012, updated daily. (2002).
34 Gfeller, G. *et al.* Representativeness and seasonality of major ion records derived from NEEM firn cores. *The Cryosphere* **8**, 1855-1870 (2014).
35 Zennaro, P. *et al.* Fire in ice: two millennia of boreal forest fire history from the Greenland NEEM ice core. *Clim. Past* **10**, 1905-1924 (2014).
36 Kang, J.-H. *et al.* Mineral dust and major ion concentrations in snowpit samples from the NEEM site, Greenland. *Atmos. Environ.* **120**, 137-143 (2015).
37 Zhao, X. *et al.* A case study of a transported bromine explosion event in the Canadian high arctic. *J. Geophys. Res. Atmos.* **121**, 457-477 (2016).
38 Belt, S. T. & Müller, J. The Arctic sea ice biomarker IP25: a review of current understanding, recommendations for future research and applications in palaeo sea ice reconstructions. *Quaternary Sci. Rev.* **79**, 9-25 (2013).
39 Dahl-Jensen, D. *et al.* Past temperatures directly from the Greenland ice sheet. *Science* **282**, 268-271 (1998).
40 Kinnard, C. *et al.* Reconstructed changes in Arctic sea ice over the past 1,450 years. *Nature* **479**, 509-512 (2011).
